# Jacobson and Truax Method: evaluation of the clinical effectiveness of a home care program after prostatectomy^1^

**DOI:** 10.1590/1518-8345.2249.3003

**Published:** 2018-05-17

**Authors:** Luciana Regina Ferreira Pereira da Mata, Mariana Ferreira Vaz Gontijo Bernardes, Cissa Azevedo, Tânia Couto Machado Chianca, Maria da Graça Pereira, Emilia Campos de Carvalho

**Affiliations:** 2 Sc.D., Adjunct Professor III, Nursing, Escola de Enfermagem da Universidade Federal de Minas Gerais, Belo Horizonte, MG, Brazil.; 3 Stomatherapy Nursing Specialist - Universidade Cândido Mendes. Sc.M., Escola de Enfermagem da Universidade Federal de Minas Gerais, Belo Horizonte, MG, Brazil.; 4 Sc.M., Doctor Degree Student, Escola de Enfermagem da Universidade Federal de Minas Gerais, Belo Horizonte, Minas Gerais, Brazil. Sponsorship: Coordenação de Aperfeiçoamento de Nível Superior - CAPES, demanda social.; 5 Sc.D., Full Professor, Nursing, Escola de Enfermagem da Universidade Federal de Minas Gerais, Belo Horizonte, MG, Brazil.; 6 M.D., Associate Professor, Applied Psychology, Universidade do Minho, Braga, Cávado, Portugal.; 7 Sc.D., Full Professor, Enfermagem Fundamental, Escola de Enfermagem da Universidade de São Paulo, Ribeirão Preto, SP, Brazil.

**Keywords:** Nursing, Prostatectomy, Health Education, Clinical Trial, Clinical Nursing Research

## Abstract

**Objective:**

to exemplify the applicability of the Jacobson and Truax Method in a nursing intervention study that analyzed the effectiveness of a home care teaching program after radical prostatectomy.

**Method:**

this is a descriptive study concerning the applicability of the Jacobson and Truax Method in the data analysis of a clinical trial. The intervention consisted of a teaching program for hospital discharge after radical prostatectomy through oral guidance, writing, and telephonic reinforcement. Thirty-four men participated in the intervention group and 34 men participated in the control group. A reliable index of change and clinical significance was calculated for the knowledge variable in both groups. Scatterplots were presented to demonstrate the effectiveness of the method.

**Results:**

for 30 individuals in the intervention group, the intervention presented clinically relevant change than in knowledge. In the control group, none of the 34 individuals presented clinical significance of the results related to this variable, that is, the statistical significance identified by the inferential tests did not have clinically relevant changes in the knowledge variable.

**Conclusion:**

the educational intervention carried out through the combination of oral, written and telephone counseling was shown to be clinically effective in improving knowledge about home care.

## Introduction

Nurses are considered the front-line care professionals and they have great influence on the experiences and outcomes of the clinical evolution of patients. In this sense, the number of studies in the nursing area associated with the actions of nurses and their impact on clinical outcomes^1^ has significantly increased in recent years.

However, this number of clinical trials in nursing is still incipient, mainly due to the recent area of activity of the profession and high cost for development^2^. However, it is known that it is an area with important development potential, capable of expanding the professional clinical practice to contribute to the improvement of health care for the population. Authors point to a predominance of this method of study in the area of adult and female health, and a smaller number in the health of the child, elderly, worker, and neonatology^3^.

Regarding methods of analysis of results, there has been an investment in methods and criteria to evaluate the effectiveness of clinical practices in the last decades^3^ to identify the really effective procedures, with questions related to the variability of results among participants of the same intervention and the clinical effect of it. Besides identifying the statistically significant differences between individuals, there is a concern to verify the significance and adaptive functionality of the changes brought about by the intervention, which is not necessarily guaranteed by the statistical significance^4^ ^-^ ^5^.

The effectiveness of an intervention, whether educational or clinical, implies gathering evidence on the internal validity of the interventions (degree that the results can be attributed to the procedures used) and on social or external validity (impact on the daily functioning of the individual, generalization for other environments or population, cost-benefit ratio)^6^.

In this perspective, several proposals for the analysis of the effectiveness of the interventions, mainly directed to the investigation of the clinical significance of the results obtained^7^ appeared. Among them, there are Jacobson and Truax (JT) highlighted^8^, known as the JT Method. This method articulates the analysis of clinical significance with the verification of the reliability of the changes obtained^8^. It can be used as a complement to the analysis of statistical significance when working exclusively with numerical scales. Also, it is considered an alternative method when the number of subjects makes inferential statistical analysis impossible^5^ ^-^ ^6^.

Practically, the JT method proposes a comparative analysis of pre and post intervention scores to decide if the differences between the participants represent reliable changes and if they are clinically relevant^4^ ^-^ ^7^. Therefore, this method tries to answer two questions: did the gains of the individual go beyond a mere oscillation (positive or negative) due to the measurement error? What is the final condition of the individual in relation to the scores of non-clinical reference groups? Thus, data analysis using the JT Method implies two complementary procedures: the calculation of the reliability of the changes that occurred between the pre-assessment and the post-intervention evaluation, described in terms of a Reliable Change Index (RCI), and the analysis of the clinical significance of these changes^5^ ^-^ ^7^.

This study shows the application of this method in a clinical study that evaluated the effectiveness of a teaching program for the home care of patients submitted to radical prostatectomy, to evaluate the operationalization and applicability of the JT Method in nursing intervention research, from the dimensions of self-efficacy, anxiety, psychological morbidity (anxiety plus depression), satisfaction and knowledge.

After radical prostatectomy, patients may have different symptoms, such as fatigue, decreased physical capacity, urinary tract infection and surgical incision, sexual dysfunction and urinary incontinence^9^ ^-^ ^10^. Considering these possible changes after prostatectomy, it was proposed the elaboration of a teaching program based on nursing orientations that improve the knowledge of these individuals about home care, for a greater capacity for self-care, increased satisfaction with care postoperative period and decreased psychological morbidity.

Thus, the objective of this study was to exemplify the applicability of the JT Method in a nursing intervention study that analyzes the effectiveness of the teaching program for home care after radical prostatectomy.

## Method

This is a descriptive study regarding the applicability of the JT method in the data analysis of a clinical trial.

The study was carried out in three hospitals in the interior of Minas Gerais from January 2012 to February 2013, with patients undergoing radical prostatectomy who had the following eligibility criteria: age above 18 years old, cognitive ability for participation assessed at application of the mini-mental state examination^11^, locomotor, visual, auditory and self-care skills, and telephone to follow up on the teaching program. Participants were randomly divided into two groups: Control Group (CG) and Intervention Group (IG).

The sample size was estimated considering the expected difference between CG and IG for self-efficacy, after treatment^12^, at a significance level of 5%, and power of 80%, resulting in 33 individuals in each group.

It was approved by the Research Ethics Committee (CEP) under protocol number 42/2011. The clinical trial was enrolled in the Brazilian Registry of Clinical Trials under the number: RBR-5n95rm. All the patients who accepted to participate in the study signed a free and informed consent term, in compliance with the legislation in force in the country.

The intervention consisted of a teaching program for hospital discharge, elaborated from the combination of oral orientation, writing, and telephonic reinforcement. A booklet called “Guidelines for home care: Prostate Radical Surgery” was developed, and a script based on the Theory of Self-efficacy^13^ was developed to guide the telephone calls in clarifying doubts and reinforcing the guidelines contained in the booklet, stimulating self-care.

The study was developed in four steps in a two-month follow-up. In T0, sociodemographic and clinical variables, self-efficacy, anxiety, psychological morbidity, satisfaction with post-operative care and knowledge were collected; randomization of participants in two IG (n=34) and CG (n=34) groups; and the beginning of the intervention with booklet delivery and oral guidance. In T1, the first telephone call was made between the third and fifth postoperative day; and in T2, the second telephone call was 30 days after discharge. Two months after T0, in the second medical return, the variables self-efficacy, anxiety, psychological morbidity, knowledge, and satisfaction in both groups were measured (T3). It should be emphasized that the CG continued in usual hospital discharge from the health service, without any intervention of the research.

When comparing the variables in the IG with the CG in the post-test, significant differences between the groups for satisfaction (p ≤ 0.001) and knowledge (p ≤ 0.001 ) were identified from inferential tests (parametric and non-parametric).

Thus, to verify if the dependent variables that presented statistical significance by the inferential tests also presented clinical significance, the JT Method was used. Based on the assumption that the JT Method is applicable for numerical scales, the RCI and clinical significance proposed by Jacobson and Truax^8^ were calculated for the knowledge variable in both IG and CG. The JT Method was not applied to the satisfaction variable since it was only an evaluation item with Likert type measurement.

The instrument for assessing knowledge about home care after radical prostatectomy consisted of a questionnaire elaborated by the authors, with 23 questions with “right”, “wrong” and “do not know” answers. The phrases correspond to the guidelines contained in the booklet “Guidelines for home care: Radical Prostate Surgery” and allow evaluating the knowledge that patients have about the care in the postoperative period of radical prostatectomy. For each correct answer, a point was assigned, totaling a maximum of 23 points. For wrong answers or do not know, there was no punctuation. The reliability of this instrument in the studied sample evaluated by Cronbach’s alpha was 0.71, considered as acceptable^14^.

In order, the pre and post-test scores of each individual are required and the value of the standard error of the difference to calculate RCI, according to the formula^8^:

$$\text{RCI =}\frac{\text{pos}-\text{pre}}{{\text{EP}}_{\text{dif}}}$$

EP*dif* = standard error of the difference, obtained from the formula: EPdif = SD1√2√1-r

Where: DP1 = standard deviation (group or individual); r = Reliability index of the measuring instrument (usually Conbrach’s alpha)

From the calculation of RCI, the following parameters are considered^8^: RCI greater than 1.96 is defined as Positive Reliable Change; RCI less than -1.96 is Reliable Negative Change; and RCI values between -1.96 and 1.96 are defined as Absence of Change.

Thus, any positive or negative oscillation between pre and post-test scores is classified as a reliable change if it is sufficiently robust to overcome the uncertainty associated with measurement errors or variability of the evaluated object, placing it within the confidence interval for the results obtained^6^.

For the calculation of the cut-off point of clinical significance, the method considers three criteria (A, B and C)^8^:

Criterion A: used when normative data are not available, being able to estimate mean and standard deviation based on the pre-test data of the clinical sample (or dysfunctional population) under treatment. In this case, a change is considered clinically relevant if the difference between pre and post-test is at least two standard deviations above the pre-test mean in the indicators of the skill being trained.

Criterion B: used when normative data are available on the distribution of functional population scores, a clinically relevant change is considered when the post-intervention score shifts the individual into the functional population distribution. That is, their post-test scores should be within the range starting at the cut-off point represented by the mean minus two standard deviations of that population.

Criterion C: used when normative data are available on the distribution of functional and dysfunctional population scores; a clinically relevant change should lead the individual, after the intervention, simultaneously out of the dysfunctional distribution and into the functional distribution. That is, the final score of the individual should place it above the point defined by the mean plus two standard deviations of the dysfunctional population and above also the average minus two standard deviations of the functional population.

For the delimitation of the confidence interval of clinical significance, the formula for the calculation of the standard error of measurement is used:

PC±1,96×(DP÷√n)

where: PC = Cut-off point calculated based on one of the criteria (A, B or C); SD = pre-test standard deviation of the clinical population; n = Number of participants.

The authors of the JT method^8^ use a classification, based on RCI verification and clinical significance: recovered - when met both criteria; improved - when passed RCI but not for clinical significance; unchanged - when did not meet any of the criteria; deteriorated - when went through the RCI in the sense of worsening.

The results of the study used to demonstrate the effectiveness of the JT method in the analysis of an educational intervention were organized and presented from scatterplots in which pre-test scores were plotted on the x-axis and post-test scores on the y-axis. Also, for the interpretation of the graphs, it is necessary to understand that the diagonal tracing called the bisector indicates that individuals located above it had improved due to the intervention and individuals below had worsened due to the intervention. However, for individuals located above the line or within the confidence interval (traced below and above the bisector), no statements of improvement or worsening can be made regarding the intervention.

## Results

From the use of the JT method in a clinical intervention study, consisting of a teaching program in the home care of patients submitted to radical prostatectomy, the effectiveness of its use was verified. In this study, considering the statistical significance found in the pre-test for the post-test in the CG at the level of the knowledge variable, the RCI and clinical significance were calculated for this variable in both OG and CG. The pre-test standard deviation of the GI equal to 3.5 and the GC equal to 3.2 was considered to calculate the standard error of the difference, and the reliability of the measurement instrument (Alfa de Cronbach) equal to 0.71, obtaining for the IG and CG the values of 2.524 and 2.322, respectively.

**Figure 1:**
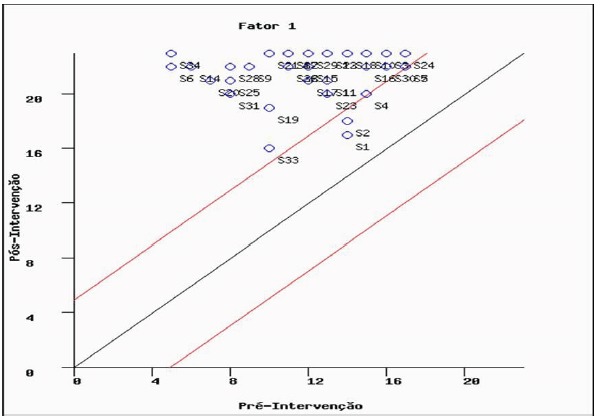
Reliable change index of the variable pre and post-test knowledge: intervention group

Thus, when calculating the difference between pre-test and post-test divided by the standard error of difference (2.524) for each individual, it was identified that only two of the 34 participants (S1 and S2) did not present a reliable change for the knowledge variable, since, according to Figure 1, they were located between the above and below the bisectors, that is, they did not improve or worsen the knowledge due to the intervention.

**Figure 2:**
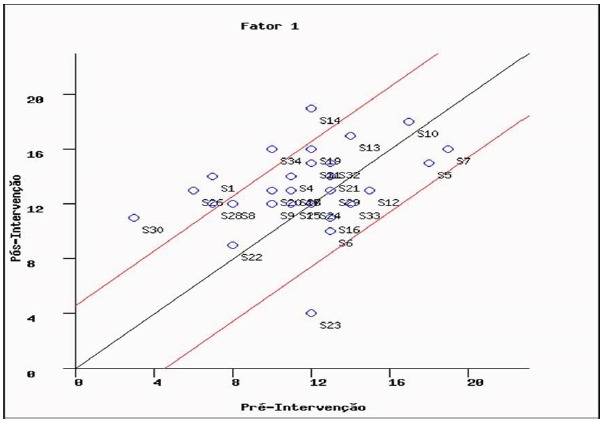
Reliable change index of the variable pre-test and post-test knowledge: control group

Regarding the CG, when calculating the difference between pre-test and post-test divided by the standard error of difference (2.322) for each individual, it was identified that one of the 34 participants (S23) presented a reliable negative change, most of them (n=28) were located between the traces above and below the bisector, that is, they did not improve or worsen the knowledge, and six of the participants (S1, S14, S26, S28, S30, S34) showed a positive change in knowledge (Figure 2).

**Figure 3:**
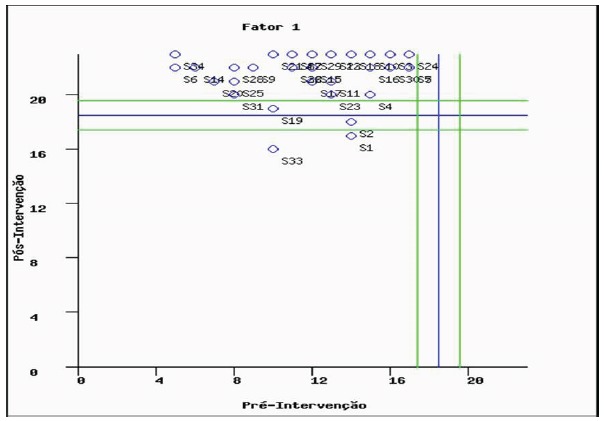
Clinical significance of the pre-test and post-test knowledge variable: intervention group

Also, the mean (M=11.47) and the standard deviation (SD=3.5) of the IG in the pre-test were considered for the calculation of the clinical significance of the knowledge variable in the IG. From this criterion, it was considered a clinically relevant change if the difference between the pre-assessment and the post-test evaluation was at least two standard deviations above the pre-test mean. The cut-off point found for clinical significance was 18.470 and the confidence interval was 1.093. Thus, in four of the 34 individuals (S1, S2, S19, S33) it was not possible to infer that the intervention presented a clinically relevant change to the knowledge variable, according to Figure 3.

**Figure 4:**
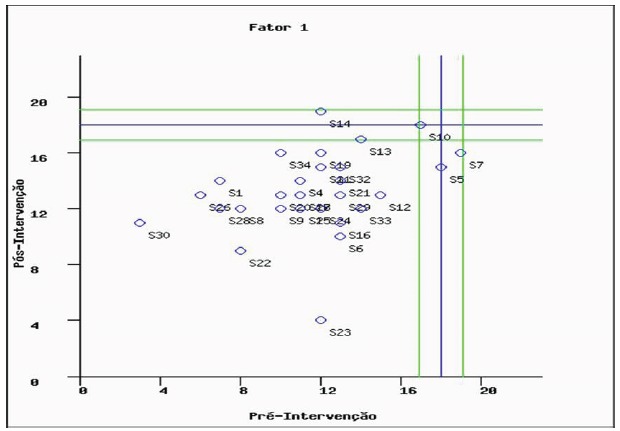
Clinical significance of the pre and post-test knowledge variable: control group

Regarding the clinical significance of the knowledge variable in the CG, the mean (M=11.56) and the standard deviation (SD=3.2) of the CG in the pre-test were considered, and the cut-off point was found for significance clinic of 18.000 and the confidence interval of 1.067. Thus, according to Figure 4, none of the 34 CG individuals presented clinical significance of the results related to knowledge, that is, the statistical significance identified by the inferential tests and the positive RCI presented by six CG individuals did not represent clinical changes knowledge level in this group.

## Discussion

Most researchers concentrate on the result being statistically significant, that is, it may not be the result of chance. However, just because the test shows that the effect of treatment is statistically significant, it does not mean that the outcome is clinically important^15^. For example, if a large sample size study has a small standard error, it is easier to find small and unimportant effects for treatment that is statistically significant^5^.

Therefore, when a clinical trial presents a statistically significant difference in its variables, one should also consider whether it is clinically important and large enough to merit a change in practice^16^ ^-^ ^17^.

Thus, it was possible to answer the two questions in this study based on RCI analyzes and clinical significance^8^. In IG, only two of the 34 individuals did not have a reliable change regarding the knowledge variable, and for only four men, it was not possible to infer that the intervention presented clinical significance for the knowledge variable. In CG, an individual presented a reliable negative change and most of them did not present a reliable change. In the CG, no clinical significance was identified for the knowledge variable in any of the individuals, that is, the statistical significance indicated by the inferential tests did not represent a clinically relevant change in the knowledge variable in the CG.

Therefore, it is suggested that patients with poor knowledge regarding the necessary care at home after surgery are subject to a negative impact on their clinical evolution, since the education of the patient has a satisfactory relation with the reduction of the occurrence of complications, satisfaction improvement, and increased capacity for care and quality of life^18^. In the context of the patient with prostatectomy, adequate knowledge allows the patient to be able to perform surgical wound care and the handling of the late bladder catheter (LBC) at home, as well as to cope with physical side effects such as urinary incontinence and the erectile dysfunction, and the consequent psychological suffering that these effects bring to men and their caregivers^19^.

The JT Method articulates the analysis of clinical significance (more focused on external validity) with verification of the reliability of the changes obtained (more related to internal validity)^4^ ^,^ ^17^. In this study, it was very important to use it as a complement to the analysis of statistical significance. From its application, it was possible to reaffirm the importance of the teaching program and its clinical significance in improving the knowledge of the 34 men who participated in the intervention, as well as the non-clinical representativeness of this variable among the CG participants.

In clinical trials, the internal validity is usually verified by inferential statistical techniques based on central (mean, median) and dispersion (standard deviation, standard error) measures of the group results. These analyses evaluate the probability of occurrence of the pre and post-test differences if they are sufficiently robust to discard the hypothesis of representing mere oscillations attributable to an error of measure and to accept that there are changes, attributable to the intervention conditions. In these designs, the external validity, mainly in terms of generalization, depends on the sampling characteristics of the IG or CG (how representative the sample is of the larger population). Therefore, such tests have little information about the clinical significance of these differences^5^ ^,^ ^16^.

In the literature, some clinical research that used the JT Method for the treatment of the data in recent years were identified. There is a study highlighted that compared the performance of the JT Method with three other alternative methods to determine which one best measured the changes in treatment ratings for substance use disorders^20^. Another study evaluated an intervention program for hypertensive patients, according to the variables knowledge, skills for self-care, therapeutic adherence, coping strategies and stress management^17^, and other researchers discussed possible statistical analyses based on the relationship between RCI and clinical significance in the context of intervention for the improvement of speech and language disorders^5^. Finally, a study that verified the use of methods to quantify the clinical significance of the change during participation in an intervention program for alcohol and drug prevention was identified^21^.

In education, the JT Method has also been applied. Researchers used it to assess the progress of medical undergraduates in learning best practices and identified the major errors made by students^16^. More specifically in special education, scholars evaluated RCI and clinical significance for the results of a group of mentally retarded adults who participated in a program to promote social and communicative skills^22^. Also in special education, the effects of a phonological remediation program with eight regular students diagnosed with Down Syndrome^23^ were verified using the JT method.

Regarding the use of clinical significance in primary nursing studies, a review of the literature with the objective of analyzing the advances of the topic in the area^24^ identified that in a sample of 261 quantitative studies published in 2016, only 33 (12.6%) reported results regarding clinical significance. In some of these 33 studies, the citation of the term clinical significance was performed without analysis basis and definition of evaluation strategy. This finding refers to the need to prioritize investigations that discuss this type of analysis in the context of nursing practices since statistical significance does not guarantee that the results are clinically meaningful, that is, they may have genuine and applicable effects on patients’ health or health care decisions^25^.

Therefore, it is expected that the JT Method will offer sufficient advantages for its use in clinical change assessment research, and that may eventually be used by other Brazilian researchers, who wish to have an objective and reliable form of evaluation of change, without disregarding the clinical relevance of the procedure.

A limitation of the study used to exemplify the JT method is the lack of validity of the questionnaire “Knowledge about home care after radical prostatectomy” by the factorial analysis, due to the small number of individuals that composed the sample, being possible only the reliability analysis of the instrument by Cronbach’s Alpha.

## Conclusion

With the use of the JT method in the analysis of the data of the exemplified clinical study and from the results found, the educational intervention carried out through the combination of oral orientation, writing and telephone follow-up was clinically effective in the scope of improvement knowledge about home care.

It is considered that this study contributes to the nursing science by proving the clinical effectiveness of the proposed intervention. It is clear the relevance of the preparation of patients for hospital discharge, mainly based on the knowledge needs about post-surgical care involving the treatment of individuals with a pathology such as cancer. It is imperative that the nurse carry out the planning and implementation of educational strategies capable of strengthening the knowledge to generate clinical impact in the reestablishment of the patient.

In the context of methodological advances, it is believed that this study also has a contribution to future clinical trials in nursing, from the presentation and application of the JT Method, still little known and disclosed in nursing.

It can be argued that the main differential of the JT Method is the possibility of analyzing individual results. That is, comparing the results of each person before and after a given intervention, even when group parameters are used for the reliability question. Thus, it is expected that this work will contribute to disseminate the potential of this method and stimulate researchers and professionals for its use in clinical nursing research.
